# The impact of scavenging air state on the combustion and emission performance of marine two-stroke dual-fuel engine

**DOI:** 10.1038/s41598-024-66826-z

**Published:** 2024-07-09

**Authors:** Hongliang Yu, Jianqun Gao, Peng Zhang, Fang Jun Han, Qizheng Yang, Bin Cui

**Affiliations:** 1 School of Ocean, Yantai University, No. 30, Qingquan Road, Laishan District, Yantai, 264005 China; 2 Yantai CIMC Raffles Offshore Limited, 70 Zhifu Island East Road, Yantai, 264000 China; 3 Marine Engineering College, Dalian Maritime University, Dalian, 116026 China

**Keywords:** Dual fuel engine, Scavenging air pressure, Scavenging air temperature, Combustion, Emission, Mechanical engineering, Diesel fuel, Natural gas

## Abstract

The scavenging process significantly affects the combustion and emission performance of marine low-speed two-stroke dual-fuel engines. Optimizing scavenging air pressure and temperature can enhance the engine's combustion efficiency and emission control performance, thereby achieving more environmentally friendly and efficient operation of dual-fuel engines. This study focuses on marine low-speed two-stroke dual-fuel engines, analyzing the effects of scavenging air pressure (3.0 bar, 3.25 bar, 3.5 bar, and 3.75 bar) and scavenging air temperature (293 K, 303 K, and 313 K) on engine performance and emission products. The results indicate that scavenging air pressure has a greater impact on engine performance than scavenging air temperature. An increase in scavenging air pressure leads to higher thermal efficiency and power. As the scavenging air pressure increases from 3 to 3.75 bar, the indicated thermal efficiency (ITE) increases from 44.02 to 53.26%, and indicated mean effective pressure (IMEP) increases by approximately 0.35 MPa. Increased scavenging air pressure improves nitrogen oxide (NOx) and hydrocarbons (HC) emissions. For every 0.25 bar increase in scavenging air pressure, NOx emissions decrease by 3.53%, HC emissions decrease by 33.35%, while carbon dioxide (CO_2_) emissions increase by 0.71%. An increase in scavenging air temperature leads to lower ITE and IMEP. As the air temperature changes from 293 to 313 K, the ITE decreases by approximately 1%, and IMEP decreases by about 0.04 MPa. Increased scavenging air temperature improves CO_2_ emissions. For every 10 K increase in the air temperature, the CO_2_ emissions decrease by 0.02%, while NOx emissions increase by 4.84%, HC emissions increase by 34.39%. Therefore, controlling scavenging air pressure is more important than scavenging air temperature in the operational management of marine two-stroke engines. Higher power and lower NOx and HC emissions can be achieved by increasing the scavenging air pressure.

## Introduction

Environmental pollution and the shortage of oil resources have become the main issues limiting the sustainable development of ships. In order to reduce harmful emissions from engines and address energy shortages, dual-fuel engines have become the primary power source for ships. Dual-fuel engines often experience unstable combustion states and high emissions of unburned methane during the combustion process. To enhance the combustion process of dual-fuel engines, many scholars have investigated the combustion and emission characteristics of dual-fuel engines^1^. Altinkurt conducted an experimental and numerical study on variable injection timing and split diesel injection for natural gas (NG) and diesel medium-speed marine engines^2^. They found that for short injection times, hydrocarbon (HC) emissions were lower under single injection than in the split-injection case, while nitrogen oxide (NOx) emissions were higher in the single injection scenario compared to the split-injection case. With a longer injection time, split injection improved the combustion efficiency. Injection timing studies were also conducted by Yang^3^. Yalong conducted a study on diesel injection timing^4^. Many scholars have investigated the effect of NG injection timing on the combustion and emission performance of a diesel/NG dual direct injection engine^5–9^. Fayad studied the clean fuel injection strategy and its comprehensive impact on combustion characteristics and particulate matter (PM)^10^. The research team also investigated the effects of fuel additives on engine performance NOx and PM emissions^11,12^. Chen optimized the combustion chamber structure and diesel/NG injection pressure strategy of the engine using a three-dimensional simulation model^13^. They found that the straight pit combustion chamber structure can improve Indicated Thermal Efficiency (ITE) and reduce carbon monoxide (CO) emissions. In the NG mixture-limited combustion mode, increasing the diesel injection pressure and NG injection pressure can promote multi-point ignition, faster flame propagation, and lower CO emissions. Gülcan also conducted a study on the influence of NG injection timing^14^. Many scholars have investigated the effect of injector structure on the combustion and emission performance of a dual fuel engine^15–18^. Dai conducted a study related to the effects of injection timing, injection pressure, and exhaust gas recirculation (EGR)^19^. Lee and Talei have investigated the effect of EGR on the combustion and emission performance of a diesel/NG engine^20,21^. Guo analyzed the performance and emission characteristics of a dual-fuel engine with various diesel injection strategies, as well as the mechanisms behind the formation of emissions (NOx, CO, HC, and soot)^22^. Hountalas utilized experimental data to compare engine performance and combustion mechanisms in both diesel and NG modes of a marine two-stroke dual-fuel engine^23^. They found higher specific fuel consumption, faster premixed and diffusion combustion, and a 6–8% shorter combustion duration in dual-fuel. Dual-fuel operating performance was generally close to that of conventional diesel, with a 1.5% lower average efficiency and reduced carbon dioxide (CO_2_) emissions. Liu compared the performance of a dual-fuel engine at high altitude using diesel and dual fuel^24^. Park also conducted a comparative study on the performance of a NG-diesel dual-fuel engine in different fuel modes^25^. A similar study was conducted by Silvagni^26^. Rochussen compared CO_2_ and methane emissions between diesel-only and NG-diesel dual fuel configurations from generalized engine test cycles of a marine dual fuel engine^27^. Kim numerically investigated the NG case with a 97% substitution rate (CNG97) using a three-dimensional computational model^28^. They found that diesel fuel could ignite CNG97 steadily with the ignition position near the center of the cylinder. Part of the NG was retained in the piston groove and top gap, significantly delaying combustion and leading to a severe phase loss of CNG97. Sattarzadeh, Park, and Lee have investigated the effect of fuel mode and substitution rates on the combustion and emission performance of engine^29–31^. Liu experimentally investigated the variation patterns of NG energy substitution rate and pilot diesel injection time under single injection strategy and split injection strategy^32,33^. The low-pressure post-injection (LPPI) strategy was proposed by Lu^34^. They compared the LPPI with the low-pressure injection (LPI) strategy. They found that LPPI increases the cylinder swirl rate to 60.7%, reduces the combustion duration by 33.6%, and extends the lean burn limit to 0.30. The NOx emissions are more than three times higher than those of LPI, which complies with the Tier III NOx emission standards.

Through the analysis of the aforementioned public literature, it can be concluded that current research on dual-fuel engines primarily focuses on small to medium-sized four-stroke engines. Limited research exists on low-speed two-stroke dual-fuel marine engines, and the influence of scavenging parameters on the performance of large low-speed two-stroke dual-fuel engines is currently a research gap. The scavenging methods, injector positions, and combustion chamber types of low-speed two-stroke dual-fuel marine engines differ from those of small to medium-sized engines. The combustion process and emission characteristics cannot be directly applied from existing small to medium-sized engines. For low-speed two-stroke dual-fuel marine engines, conducting performance studies through hundreds or thousands of experiments, as is done with small to medium-sized engines, is not feasible due to their enormous size. The research method of collecting data through experiments for low-speed two-stroke dual-fuel marine engines faces limitations in terms of location and equipment. Research on emission reduction and performance enhancement of two-stroke engines currently centered on the use of low-carbon fuels and optimizing fuel injection timing. The application of natural gas and methanol fuels in ships is well-established, while the application of ammonia fuel in ships is still in the research and development stage. Fuel injection technology for marine vessels is also becoming increasingly mature, with ships currently utilizing variable injection timing to control engine combustion. Studies on the impact of scavenging parameters on emission reduction and performance enhancement of two-stroke engines are very limited. To thoroughly analyze the impact of scavenging conditions on low-speed two-stroke dual-fuel marine engines, this study integrates numerical calculations with experiments. The study focuses on the effects of scavenging pressure and temperature on the combustion process and emissions of low-speed dual-fuel marine engines. Keeping other boundary conditions constant, this research analyzed the influence of scavenging pressure (3.0 bar, 3.25 bar, 3.5 bar, and 3.75 bar) and scavenging temperature (293 K, 303 K, and 313 K) on the combustion process of dual-fuel engines operating in diesel-ignited natural gas fuel mode.

## Research methods

### Research target

This study is based on an S50ME-GI type low-speed engine. The engine is a two-stroke, turbocharged, water-cooled engine with an open combustion chamber. Calculated data of engine are shown in Table 1. NG is injected by high-pressure direct injection and ignited by direct in-cylinder diesel fuel injection in front of the top dead center (TDC). The diesel particulate emission collection equipment uses HOR1BAMDLT-1302TMA, the exhaust analyzer uses HORJBAMEXA-1600DS, and the other test equipment is shown in Table 2.

**Table 1:** Calculated data of engine.

Name	Data	Name	Data
Bore × Stroke (mm)	500 × 2000	Method of aspiration	Turbocharger
Rated speed (r/min)	108	Rated power (kW)	8100
Diesel injection holes	5 × *Φ*1.05 mm	NG injection holes	4 × *Φ*2.2 mm
Maximum burst pressure (MPa)	17	Nominal compression ratio	15
NG injection timing (°CA)	−4 to 20	NG temperature (K)	318
NG injection pressure (MPa)	30	NG supply (g/r)	182.94
Diesel injection timing (°CA)	−6 to −2	Diesel temperature (K)	311
Diesel injection pressure (MPa)	35	Diesel supply (g/r)	7.26
Valve temperature (K)	843	Piston temperature (K)	661
Liner temperature (K)	491	Pilot diesel ratio (%)	5
Scavenging air pressure (bar)	3.34	Scavenging air temperature (K)	311

**Table 2:** Test main instruments.

Analyzer	Model	Measurement ranges	Deviation (%)
CO (ppm)	AIA 240	0 ~ 1000	0.16
CO_2_ (%)	AIA 240	0 ~ 16.0	0.12
NOx (ppm)	FAC 246	0 ~ 2000	0.11
O_2_ (%)	IMA 241	0 ~ 25	−0.10
HC (ppm)	FAC 246	0 ~ 2000	0.15
*t* (^o^C)	FC2022	0 ~ 1000	0.1
*p* (kPa)	FC2022	−50 ~ 4000	0.1
Speed (r/min)	FC 2010	0 ~ 3000	0.1
Torque (N·m)	CFSR-26	0 ~ 37,300	0.25
Fuel flow (kg/h)	FC 2210	0 ~ 1000	0.1
LNG flow (m^3^/h)	CMF 200 M	0 ~ 2000	0.2

### Modeling and validation

**Figure 1:**
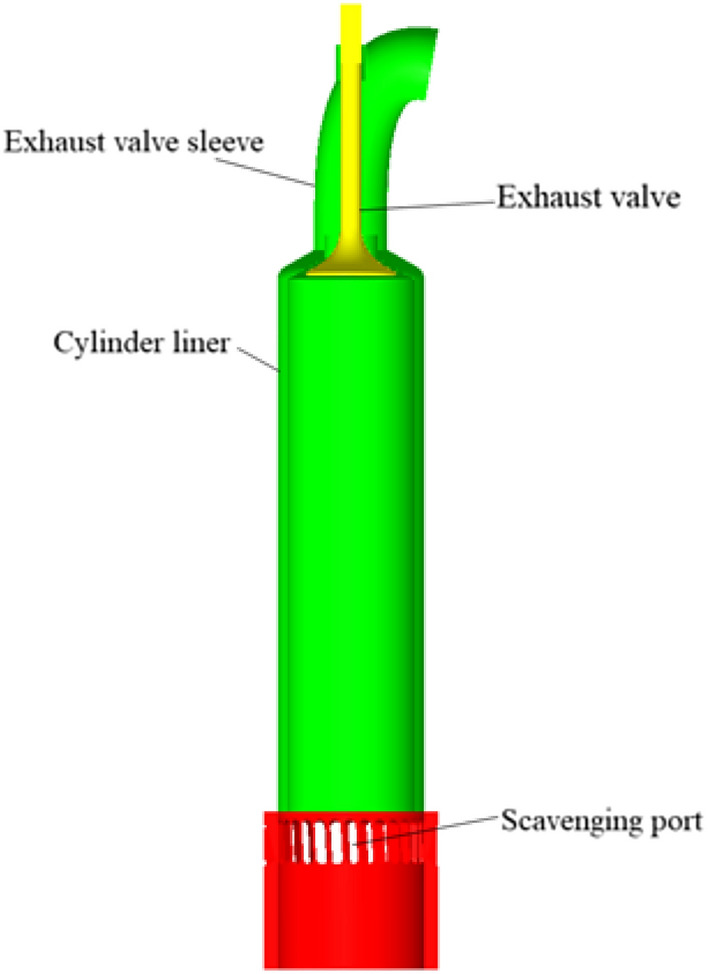
Schematic diagram of marine engine model.

**Figure 2:**
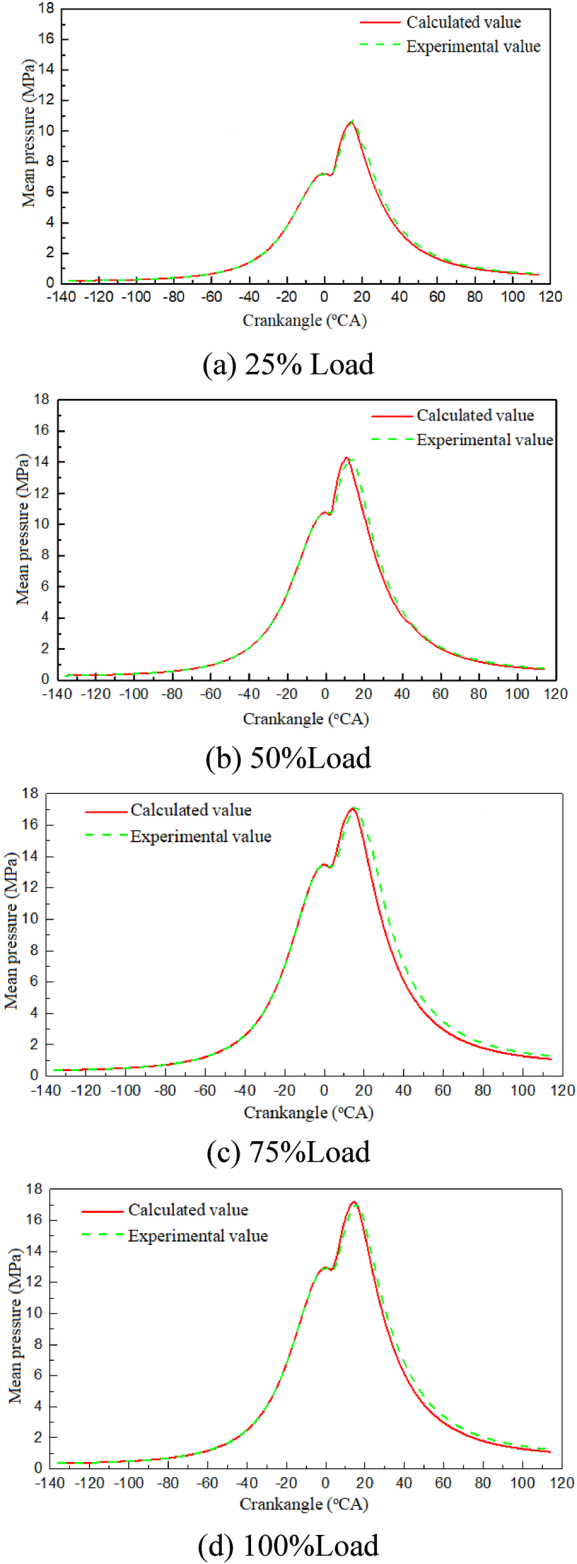
Comparison of pressure at different load.

**Figure 3:**
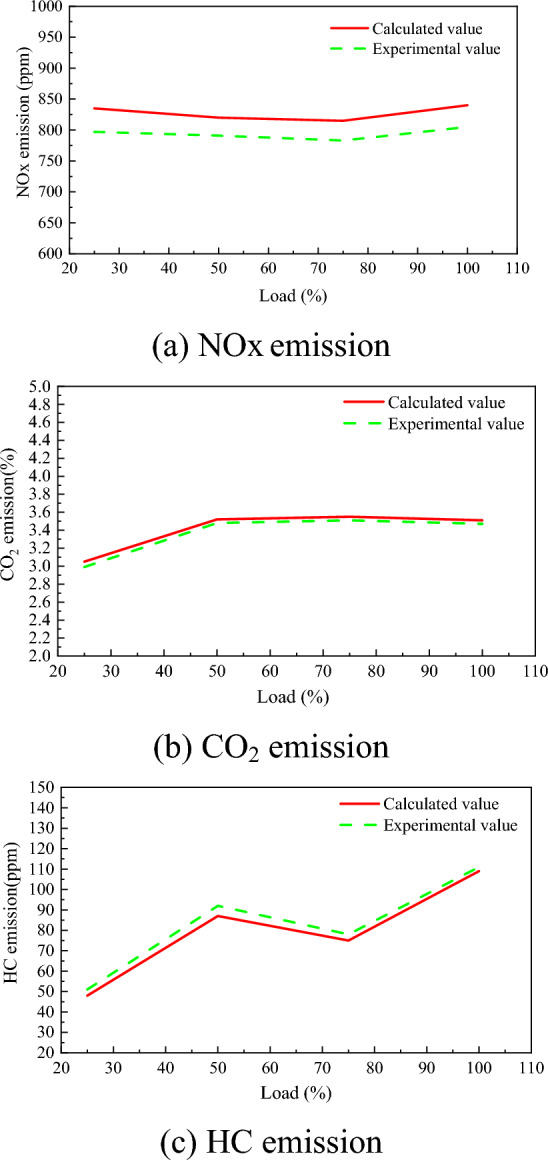
Comparison of emission at different load.

A Computational Fluid Dynamics (CFD) model of a marine dual-fuel engine (shown in Fig. 1) was developed using the three-dimensional CFD software AVL FIRE Version 8^35^ to simulate the flow, spray, and combustion processes. Using n-heptane as the surrogate fuel for diesel fuel, the reaction mechanism of methane, the primary component of NG, comprises 51 components and 252 reactions^36^. Computational analysis is conducted using a high-performance computing system equipped with a 32-core CPU and dual GPU CUDA. The number of diesel injection holes is 5, while the number of NG injection holes is 4, fanning out towards the center of the cylinder. Due to the large cylinder diameter of the marine engine, after analyzing the mesh sensitivity, the basic mesh size was set to 1 cm to ensure the accuracy and computational efficiency of the calculation. Additionally, a minimum local mesh size of 4 mm was implemented, along with adaptive refinement based on velocity and temperature. The maximum number of meshes for the calculation reached approximately 1.84 million. The calculation was performed from the moment the scavenging port was closed (−138 ℃A) to the moment the exhaust valve was opened (114 ℃A). The initial pressure in the cylinder was 3.34 bar, the initial temperature was 372 K, and the gas composition was set according to the scavenging air. Due to the large size of the two-stroke engine and space constraints, the experiment was conducted at the shipyard. First, the engine's various systems were prepared, and then the engine was started. The engine was operated at stable loads of 25%, 50%, 75%, and 100% for at least 30 min each to collect data. Each experiment took approximately 3 h to complete. Figure 2 shows the comparison of indicator diagram pressures of the engine at 25%, 50%, 75%, and 100% loads. Upon comparing the pressure curves of each load, a deviation was observed between the calculated values and the experimental values. Calculations deviated from experimental data due to errors in the experimental environment and initial calculation information, leading to uncertainties in the comparative results. This uncertainty arises primarily from two factors. First, the air density used by the engine for scavenging in the experiment is influenced by the temperature and humidity of the laboratory environment, which cannot be consistent with the ideal air in the calculations. Second, the experiment used a blend of diesel and natural gas, while the calculations simulated diesel by using n-heptane and natural gas by using methane for the engine combustion process. Despite the deviations, the overall linear trend of the calculated values was consistent with the experimental values, indicating that the calculation model accurately reflected the combustion process characteristics of the engine. Figure 3 shows that the calculated values of CO_2_, NOx, and HC were in good agreement with the experimental values in the overall linear trend, indicating that the emission model could accurately predict the emission characteristics of diesel-ignited natural gas combustion conditions.

## Results and discussion

### Impact of scavenging air pressure

Under the conditions of a diesel energy ratio of 5% and scavenging air temperature of 311 K, calculations were conducted for in-cylinder combustion processes at different scavenging air pressures (3.0 bar, 3.25 bar, 3.5 bar, and 3.75 bar).

#### Combustion process

**Figure 4:**
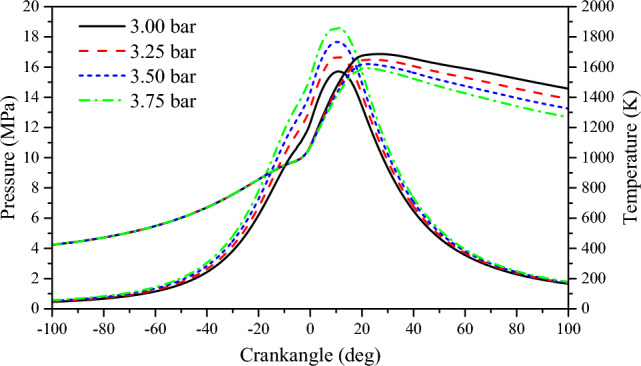
The impact of scavenging air pressure on pressure and temperature in cylinder.

**Figure 5:**
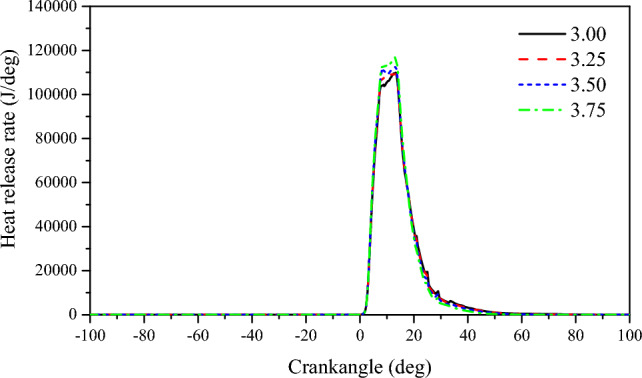
The impact of scavenging air pressure on heat release rate.

The increase in scavenging air pressure leads to a longer period of residual gas ignition in the cylinder, resulting in a larger amount of residual gas remaining in the cylinder. Once ignition occurs, most of the residual gas starts to burn. At this point, the piston is close to TDC, causing the pressure in the cylinder to deviate from the compression line and rapidly reach the maximum explosion pressure. A shorter combustion delay period is beneficial for combustion near the top dead center^37^. From Fig. 4, it can be observed that as the scavenging air pressure increases continuously, the pressure inside the cylinder gradually rises, and the curves become steeper. The maximum explosion pressure in the cylinder gradually increases with the increase in scavenging air pressure. For every 0.25 bar increase in scavenging air pressure, the peak pressure in the cylinder increases by 5.87%. Although the overall change in peak pressure is not significant, as the scavenging air pressure increases, it moves towards TDC, indicating a shorter combustion period. At 3.0 bar, the combustion period is 12.8°CA; at 3.25 bar, it is 12.5°CA; at 3.5 bar, it is 11.5°CA; and at 3.75 bar, it is 11.3°CA. As the scavenging air pressure increases, the combustion period decreases from 12.8 to 11.3°CA, resulting in a faster combustion rate in the cylinder, higher combustion quality, and ultimately an improvement in engine power. Figure 4 illustrates that as scavenging air pressure increases, the highest temperature inside the cylinder decreases. For every 0.25 bar increase in scavenging air pressure, the highest temperature inside the cylinder decreases by 1.89%. The highest temperature inside the cylinder moves towards the top dead center, indicating that the combustion delay period shortens. At 3.0 bar, the combustion delay period is 7.25°CA; at 3.25 bar, it is 7°CA; at 3.5 bar, it is 5.75°CA; and at 3.75 bar, it is 5.75°CA. Analyzing the reasons for this phenomenon reveals that with increased scavenging air pressure, more fresh air enters the cylinder during the scavenging process, thereby as the scavenging pressure increases, combustion is more rapid and the heat release rate is faster (Fig. 5). The scavenging air temperature is much lower than the temperature of the exhaust gas inside the cylinder, which further aids in cooling the cylinder through scavenging. Additionally, after the highest temperature inside the cylinder occurs, as the scavenging air pressure increases, the cylinder temperature decreases further.

**Figure 6:**
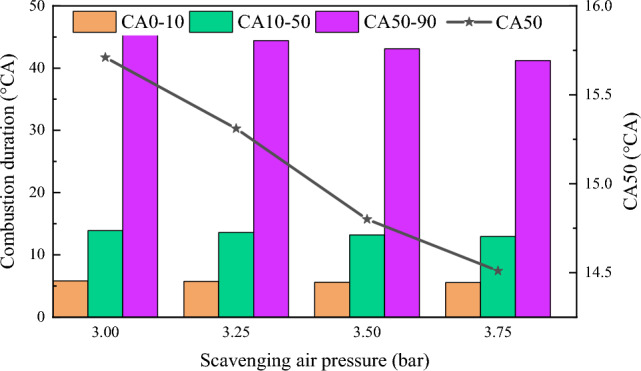
Combustion duration and center of combustion under scavenging air pressure.

Different combustion durations and combustion centers under various scavenging air pressures are shown in Fig. 6. Here, the combustion duration is divided into three stages: the first stage is the flame development period (CA0-CA10), from ignition to 10% of fuel heat release; the second stage is the first half of the main combustion period (CA10-CA50), where the fuel heat release increases from 10 to 50%; the third stage is the second half of the main combustion period (CA50-CA90), where the fuel heat release increases from 50 to 90%^38^. During the scavenging process, the air pressure increases from 3 to 3.75 bar. The combustion center temperature changed from 15.71 to 14.51℃A, moving towards TDC. The flame development period, the first half of the main combustion period, and the second half of the main combustion period all exhibit a decreasing trend. The rate of decrease in the first half of the combustion period is less than that in the second half. The main reason analyzed was the increase in scavenging air pressure, which elevated the temperature and pressure at the end of the compression stroke. This resulted in faster diesel ignition and also increased the flame propagation rate of natural gas. Consequently, this led to the early formation of the combustion center^39^.

To further analyze the impact of dual-fuel engine combustion characteristics and its effects on performance, indicated thermal efficiency (ITE), thermodynamic efficiency (TE) and indicated mean effective pressure (IMEP) are introduced for a quantitative analysis of energy conversion efficiency. The parameters are defined as follows^40^,

1 $${\eta }_{i}=\frac{{W}_{i}}{Q}\times 100\%$$

2 $${\eta }_{td}=\frac{{W}_{i}}{{Q}_{a}}\times 100\text{\%}$$

3 $$Q\, = \,m_{{{\text{Fuel}}}} \,{\text{LHV}}_{{{\text{Fuel}}}} + m_{{{\text{Diesel}}}} \,{\text{LHV}}_{{{\text{Diesel}}}}$$

In the equation, *Q*_a_ stands for cumulative heat release during the cycle; *Q* is the theoretical fuel release during the cycle; η_*i*_ denotes ITE; *W*_*i*_ is the indicated work during the cycle; η_*td*_ is the TE; m represents the mass of each fuel; LHV represents the lower heating value of each fuel.

**Figure 7:**
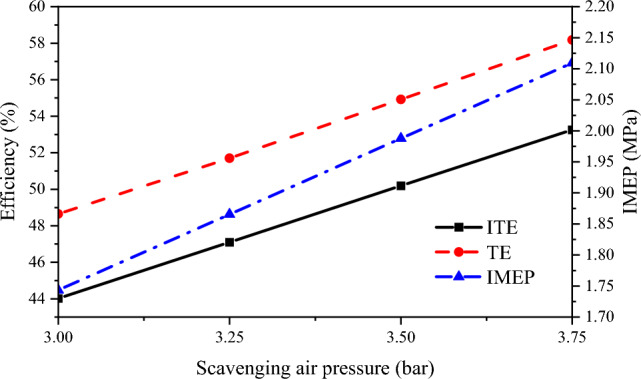
ITE, TE and IMEP under different scavenging air pressure.

The changes in TE, ITE, and IMEP under different scavenging air pressures are shown in Fig. 7. As the scavenging air pressure increases from 3 to 3.75 bar, TE, ITE, and IMEP all show an increasing trend. TE increases from 48.65 to 58.18%, ITE increases from 44.02 to 53.26%, and IMEP increases by approximately 0.35 MPa. With the increase in scavenging air pressure, the total mass of air in the cylinder increases, which results in a higher combustion velocity. This results in higher TE and ITE and consequently increasing the IMEP. As scavenging pressure increases, the cylinder stagnation period lengthens, causing a higher accumulation of natural gas in the cylinder. When ignited by diesel fuel, a substantial amount of natural gas begins to burn vigorously near the upper endpoint. This leads to a rise in IMEP and, concurrently, an increase in TE.

#### Flame propagation velocity

The flame propagation velocity in the cylinder can be calculated by measuring the distance the flame boundary moves within a unit of time. By capturing instantaneous images within the cylinder for a specific time interval, segmenting the flame boundary in image processing, and identifying the displacement coordinates of the flame boundary points A (*x*_*i*_, *y*_*i*_), it is possible to calculate the distance (∆*l*) of consecutive movements of the boundary points^41^.

4 $$\Delta l=\sqrt{{({x}_{i+1}-{x}_{i})}^{2}+{({y}_{i+1}-{y}_{i})}^{2}}$$

5 $$v=\Delta l/\Delta t$$

**Figure 8:**
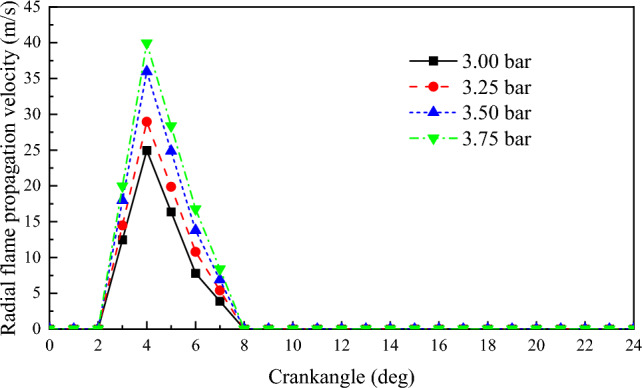
The impact of scavenging air pressure on flame propagation velocity in the radial.

**Figure 9:**
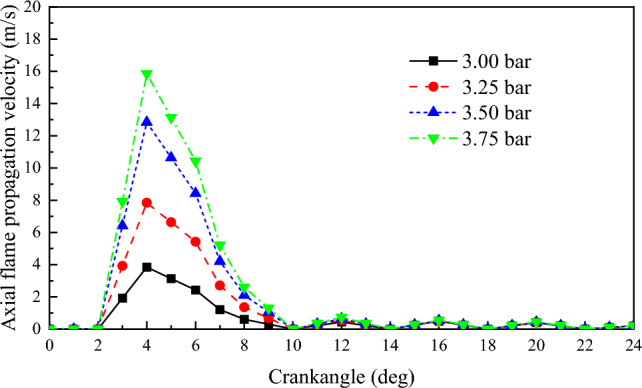
The impact of scavenging air pressure on flame propagation velocity in the axial.

∆*l* is the distance of consecutive movements of the boundary points. v is the flame propagation velocity. ∆*t* is the time interval between two consecutive movements of the flame boundary point A. Figures 8 and 9 depict the radial and axial flame propagation velocity inside the cylinder under various scavenging air pressures. With the increase in scavenging air pressure, both the radial and axial flame propagation velocity increase. This indicates that the higher scavenging air pressure enhances the combustion velocity of NG, thereby improving the engine's explosive pressure. For every 0.25 bar increase in scavenging air pressure, the radial flame propagation velocity increases by 6.1 m/s, and the axial flame propagation velocity increases by 4.2 m/s.

#### Combustion interruption

**Figure 10:**
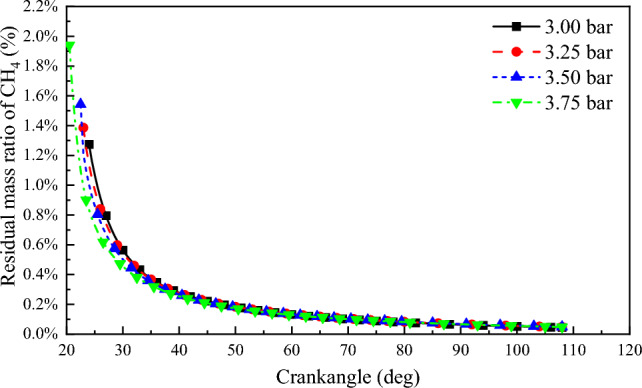
Residual mass ratio of CH_4_ after combustion end point.

Under different scavenging air pressures, the residual methane (CH_4_) mass in the cylinder after combustion endpoint is shown in Fig. 10. It can be observed from the graph that as scavenging air pressure increases, the residual CH_4_ in the cylinder decreases after combustion. At scavenging air pressures of 3.00 bar, 3.25 bar, 3.5 bar, and 3.75 bar, the combustion endpoints are 24 °CA after top dead center (ATDC), 23 °CA ATDC, 22.5 °CA ATDC, and 20.5 °CA ATDC respectively. The corresponding residual CH_4_ mass ratios in the cylinder at each combustion endpoint are 1.27%, 1.38%, 1.54%, and 1.94%, respectively. The residual CH_4_ mass ratios in the cylinder at the start of the exhaust valve for each scavenging air pressure are 0.037%, 0.039%, 0.040%, and 0.057%, respectively.

**Figure 11:**
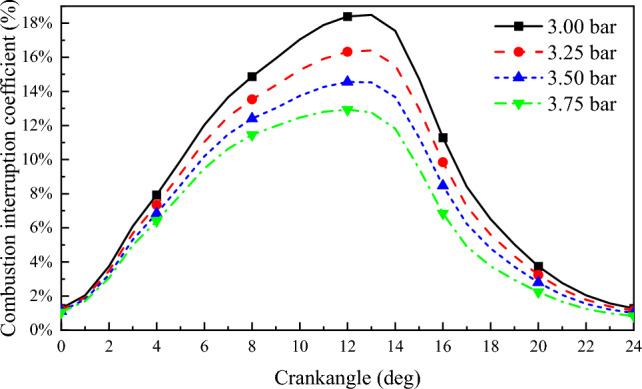
Combustion interruption coefficient.

Figure 11 shows the variation of combustion interruption coefficient with crank angle at different scavenging pressures. It can be seen from the figure that the combustion interruption coefficient decreases with increase in scavenging pressure. The peak value of combustion interruption coefficient decreases from 18.49% at 3 bar to 12.93% at 3.75 bar. For every 0.25 bar increase in scavenging air pressure, the combustion interruption factor decreases by 1.85%. Residual methane is mainly influenced by the flame propagation rate and combustion reaction rate in the cylinder. The higher the air volume in the cylinder, the faster the premixed combustion rate and the lower the methane combustion interruption factor^42^.

#### Emission

**Figure 12:**
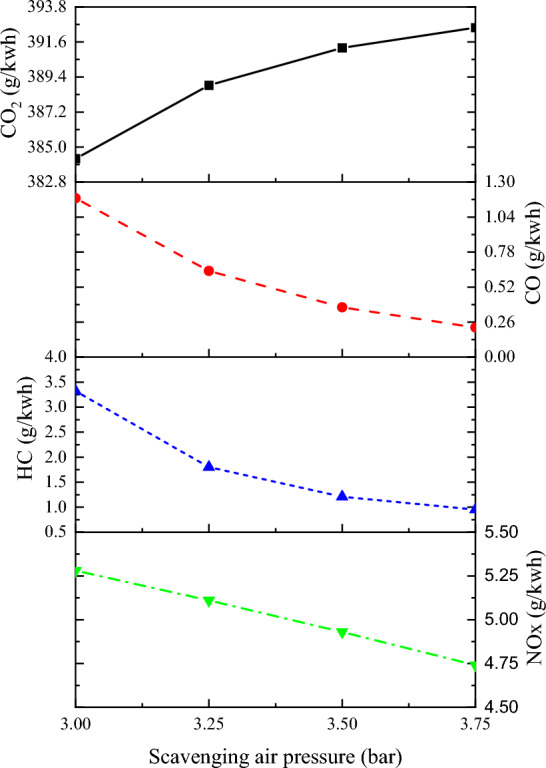
The impact of scavenging air pressure on emission.

In marine engines, fuel combustion accounts for 90% of the NOx emissions. In dual-fuel engines using natural gas and diesel fuel, NOx and methane are the most common harmful emissions. These engines produce little carbon smoke and sulfur oxides. Therefore, the primary focus was to investigate the effect of scavenging air pressure on CO_2_, CO, HC, and NOx emissions in marine dual-fuel engines. As shown in Fig. 12, an increase in scavenging air pressure results in a higher oxygen content in the engine cylinder, leading to better and faster combustion. This results in reduced CO and HC emissions and increased CO_2_ emissions. For every 0.25 bar increase in scavenging air pressure, CO emissions decrease by 42.83%, HC emissions decrease by 33.35%, and CO_2_ emissions increase by 0.71%. With the increase of scavenging pressure, the NOx emission shows a decreasing trend. For every 0.25 bar increase in scavenging air pressure, NOx emissions decreased by 3.53%. The main reason for this is that the increase in scavenging air pressure reduces the average temperature in the cylinder, shortens the time between fast and slow combustion, and reduces the temperature and time at which nitrogen remains in the hot, oxygen-rich environment. This reduces the production of NOx.

### Impact of scavenging air temperature

Under the conditions of a diesel energy ratio of 5% and scavenging air pressure of 3.34 bar, calculations were conducted for in-cylinder combustion processes at different scavenging air temperatures (293 K, 303 K, and 313 K).

#### Combustion process

**Figure 13:**
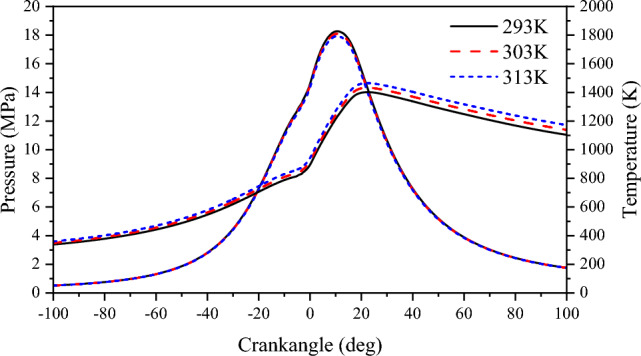
The impact of scavenging air temperature on pressure and temperature in cylinder.

**Figure 14:**
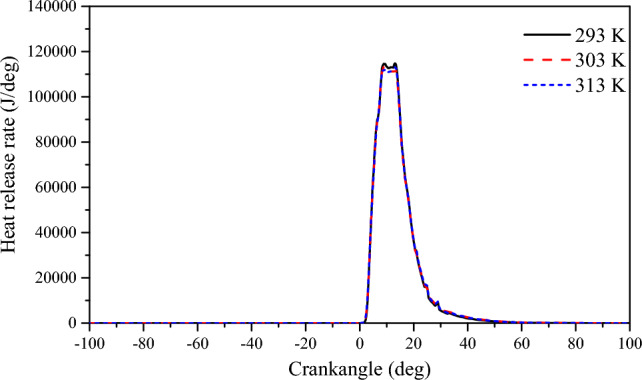
The impact of scavenging air temperature on heat release rate.

Figure 13 shows the effect of scavenging air temperature on cylinder pressure. As the scavenging temperature increases, the average pressure in the cylinder decreases, but not by much. When the scavenging air temperature is 293 K, the peak average pressure in the cylinder is 18.28 MPa. When the scavenging air temperature is 303 K, the peak average pressure in the cylinder is 18.09 MPa. When the scavenging air temperature is 313 K, the peak average pressure in the cylinder is 17.93 MPa and the peak pressure is shifted backward to prolong the combustion cycle. Increasing the scavenging air temperature increases the mixture movement rate, but it also reduces the amount of fresh air entering the cylinder, which decreases the combustion response of the NG. Under the same stoichiometric combustion conditions, the oxygen concentration in the combustible mixture of premixed combustion is proportionally reduced. This results in a slow spread of the NG thin combustion flame after ignition, which leads to lower cylinder pressure during combustion. The effect of scavenging air temperature on the cylinder temperature can be observed in Fig. 13. It is clear that an increase in the scavenging air temperature increases the initial heat value in the cylinder, leading to an increase in the cylinder temperature. When the scavenging air temperature is 293 K, the maximum temperature inside the cylinder reaches 1402 K. When the scavenging air temperature is 303 K, the maximum temperature inside the cylinder reaches 1433 K, and when the scavenging air temperature is 313 K, the maximum temperature inside the cylinder reaches 1465 K. Although the increase in scavenging temperature caused an increase in the cylinder temperature, the increase in scavenging temperature caused an increase in the volume of scavenging air per unit mass, resulting in a decrease in the amount of fresh air in the cylinder, which led to a decrease in the combustion reaction rate of the natural gas, and the heat release rate curve also showed a trend of decreasing with the increase in scavenging temperature (Fig. 14).

**Figure 15:**
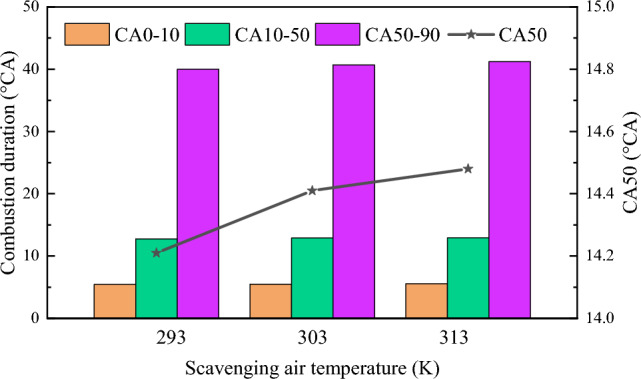
Combustion duration and center of combustion under scavenging air temperature.

Figure 15 shows the combustion duration at different scavenging air temperatures. The flame development time is essentially the same at different scavenging air temperatures, about 5.5^o^CA. The difference in the first half of the main combustion period is also not significant, about 12^o^CA. During the second half of the combustion period, the temperature of the scavenging air increased by about 2 K. Overall, the combustion of the dual-fuel natural gas/diesel engine showed a gradual downward trend. This is mainly due to the increased diffusion of natural gas molecules in the cylinder as the scavenging air temperature increases. In addition, the cylinder diameter of the marine engine is larger and it takes longer for the natural gas to reach the lean ignition limit. This results in slower combustion and delayed appearance of the combustion center.

**Figure 16:**
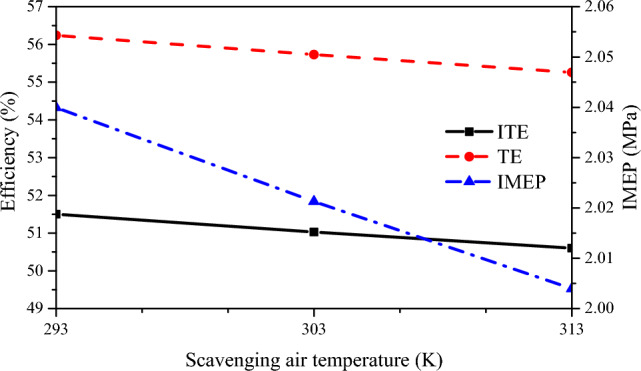
ITE, TE and IMEP under different scavenging air temperature.

Figure 16 shows TE, ITE, and IMEP at different scavenging air temperatures. as the scavenging air temperature increased, ITE, TE, and IMEP decreased to varying degrees, with ITE and TE showing similar decreasing trends. During the scavenging process, as the air temperature went from 293 to 313 K, TE decreased by about 1.5%, ITE decreased by about 1%, and IMEP decreased by about 0.04 MPa. As the scavenging air temperature increases, the oxygen content in the cylinder decreases, thus limiting the rate of combustion. This results in a decrease in ITE, TE and IMEP. Therefore, special attention must be paid to the cooling effect of the turbocharger during engine operation to ensure that the engine operates at the lowest possible scavenging air temperature.

#### Flame propagation velocity

**Figure 17:**
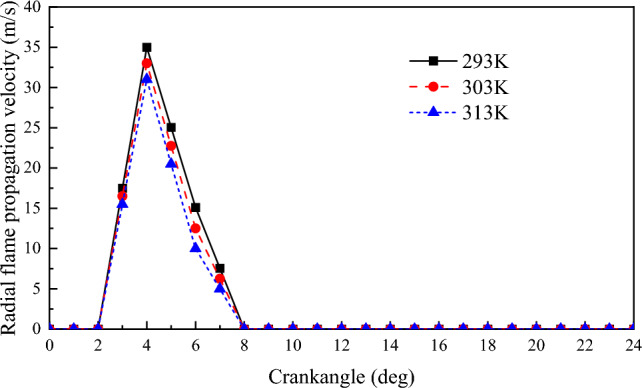
The impact of scavenging air temperature on flame propagation velocity in the radial.

**Figure 18:**
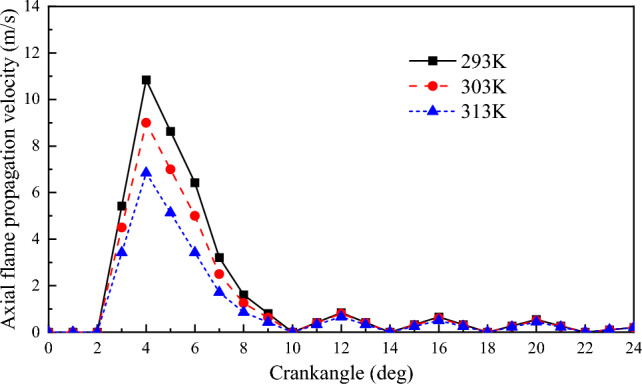
The impact of scavenging air temperature on flame propagation velocity in the axial.

Figures 17 and 18 show the radial and axial flame propagation velocities for different scavenging air temperatures. Both radial and axial flame propagation velocities tend to decrease as the scavenging air temperature increases. As the scavenging air temperature increases, the molecular diffusion of the substance in the cylinder increases, resulting in faster formation of the initial NG combustible mixture. This results in a more stable ignition of the natural gas in the cylinder^43^. However, due to the limitations imposed by the intake air volume on the thin combustion of the NG and the limitations imposed by the NG itself on the premixing time, the radial and axial flame propagation of the NG decreases rather than increases as the temperature rises. For every 10 K increase in scavenging air temperature, the radial flame propagation velocity decreases by 2 m/s, and the axial flame propagation velocity also decreases by 2 m/s.

#### Combustion interruption

**Figure 19:**
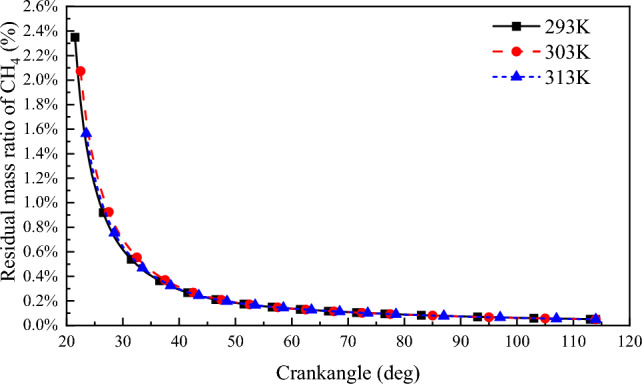
Residual mass ratio of CH_4_ after combustion end point.

The mass of CH_4_ remaining in the cylinder at the end of combustion at different scavenging air temperatures is shown in Fig. 19. From the figure, it can be seen that the CH_4_ remaining in the cylinder at the end of combustion increases with the increase of the scavenging air temperature. The flame propagation speed of lean NG combustion decreases with the increase of the scavenging air temperature, resulting in the prolongation of NG combustion duration in the cylinder. The combustion termination points at scavenging air temperatures of 293 K, 303 K, and 313 K were 21.5^o^CA ATDC, 22.5^o^CA ATDC, and 23.5^o^CA ATDC, respectively, and at each combustion termination point, the residual CH_4_ mass percent in the cylinder was 2.35%, 2.07%, and 1.56%, respectively. The lower scavenging air temperatures resulted in higher air–fuel ratios, faster combustion of the nitrogen-poor mixture, earlier combustion termination, and an engine combustion cycle that approached the ideal constant-capacity cycle, resulting in improved cycle thermal efficiency.

**Figure 20:**
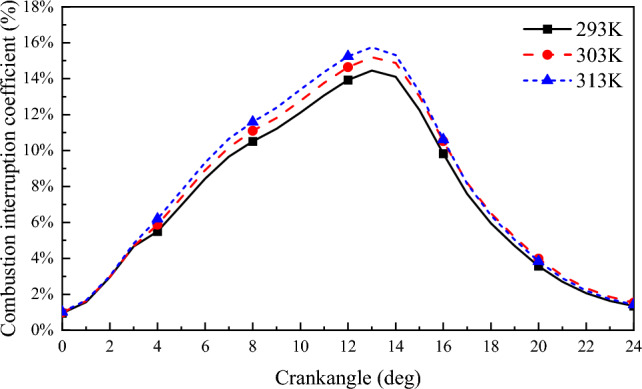
Combustion interruption coefficient.

Figure 20 shows the variation of combustion interruption coefficient with crank angle at different scavenging temperatures. It can be seen from the figure that the combustion interruption coefficient decreases as the scavenging air temperature decreases. The peak value of combustion interruption coefficient decreases from 15.74 to 14.45% during the transition of scavenging air temperature from 313 to 293 K. For every 10 K decrease in scavenging air temperature, the combustion interruption factor decreases by 0.43%. The decrease in scavenge air temperature increases the oxygen content of the scavenge air, which stabilizes the ignition of the natural gas, accelerates flame propagation, and reduces natural gas blowout and misfire in the cylinder, thus reducing the combustion interruption factor of the natural gas.

#### Emission

**Figure 21:**
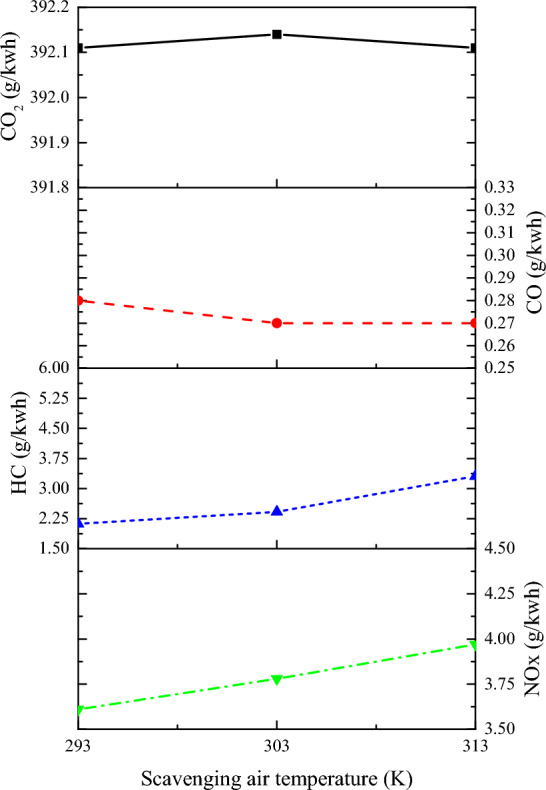
The impact of scavenging air temperature on emission.

From Fig. 21, it can be observed that as the scavenging air temperature increases, emissions of CO, HC, and NOx increase, while CO_2_ emissions decrease. For every 10 K increase in scavenging air temperature, NOx emissions increase by 4.84%, CO emissions increase by 2.51%, HC emissions increase by 34.39%, and CO_2_ emissions decrease by 0.02%. NOx in marine engine exhaust NOx is a general term for various oxides of nitrogen, which include NO, NO_2_, NO_3_, N_2_O, N_2_O_3_, N_2_O_4_, N_2_O_5_, etc. In ship engines, approximately 90% to 95% of the nitrogen oxides emitted from the exhaust pipe are NO, with a small amount of NO_2_, and other components can be neglected. For ship engines, the primary concern is the emission of NO. Since ship engine fuel contains only trace amounts of nitrogen, the formation of NO is minimal and not significant enough to cause notable NO emissions. The NO in ship engine exhaust is mainly formed by the reaction of nitrogen and oxygen in the air during high-temperature combustion, which produces thermal nitrogen oxides^44^. The generation of NO sharply increases exponentially with temperature. When the temperature is below 1800 K, the rate of NO generation is very low; it reaches a high rate at 2000 K. It can be roughly assumed that for every 100 K increase in temperature, the rate of NO generation almost doubles. Additionally, an increase in oxygen concentration also leads to an increase in NO production. Since the generation of NO is slower compared to the combustion reaction of the fuel, only a small portion of NO is produced in the thin flame reaction zone, while most NO is generated in the post-flame burned gas. If the reactants do not stay in the high-temperature environment long enough, NO does not reach equilibrium content, leading to reduce NO emissions. Therefore, the three main factors determining the rate of NO generation during the combustion process of ship engines are high temperature, oxygen-rich conditions, and the duration of nitrogen and oxygen staying at high temperatures. An increase in the temperature inside the combustion chamber, an increase in oxygen concentration, and a longer residence time of the gas in the high-temperature zone all contribute to an increase in NO. The increase in scavenging air temperature raises the average temperature inside the cylinder, leading to a high-temperature environment for NOx generation, consequently resulting in increased NOx emissions. Due to the elevated scavenging air temperature, the actual fresh air intake into the cylinder decreases. This results in a more diluted oxygen concentration in the combustible mixture, which slows down the propagation of premixed combustion flames. Consequently, it causes severe combustion interruption in the cylinder, leading to increased CO and HC emissions while reducing CO_2_ emissions. Therefore, during the operation of marine low-speed two-stroke dual-fuel engines, it is advisable to appropriately increase the inter-cooling intensity to achieve lower scavenging air temperatures. This can help in reducing HC and NOx emissions.

## Conclusions

The state of the scavenging air has an effect on both engine combustion emissions. The scavenging air pressure has a greater impact on engine performance than scavenging air temperature. An increase in scavenging air pressure leads to higher thermal efficiency and power. As the scavenging air pressure increases from 3 to 3.75 bar, the ITE increases from 44.02 to 53.26%, and IMEP increases by approximately 0.35 MPa. Increased scavenging air pressure improves NOx and HC emissions. For every 0.25 bar increase in scavenging air pressure, NOx emissions decrease by 3.53%, HC emissions decrease by 33.35%, while CO_2_ emissions increase by 0.71%. An increase in scavenging air temperature leads to lower ITE and IMEP. As the air temperature changes from 293 to 313 K, the ITE decreases by approximately 1%, and IMEP decreases by about 0.04 MPa. Increased scavenging air temperature improves CO_2_ emissions. For every 10 K increase in the air temperature, the CO_2_ emissions decrease by 0.02%, while NOx emissions increase by 4.84%, HC emissions increase by 34.39%. Therefore, controlling the scavenging air pressure is more important than regulating the scavenging air temperature in the operational management of marine two-stroke engines. Higher power output and lower levels of NOx and hydrocarbon emissions can be achieved by increasing the scavenging air pressure. While maintaining a constant scavenging air pressure, it is advisable to maximize the scavenging air cooling of the marine engine to reduce the scavenging air temperature. This will enhance the combustion emission performance of the engine. Meanwhile, future research will continue to conduct in-depth studies on marine low-speed two-stroke dual-fuel engines, with a primary focus on incorporating ammonia fuel for experimental and simulation investigations. The research will provide a theoretical basis for upgrading marine engines to save energy and reduce emissions.

## Data Availability

The data that support the findings of this study are available from Yantai CIMC Raffles Offshore Limited but restrictions apply to the availability of these data, which were used under license for the current study, and so are not publicly available. Data are however available from the authors upon reasonable request and with permission of Yantai CIMC Raffles Offshore Limited. Please contact Hongliang Yu if you need data from this study. Email: yuhongliang19852@163.com.

## Abbreviations

NG: Natural gas
HC: Hydrocarbons
NOx: Nitrogen oxide
CO: Carbon monoxide
EGR: Exhaust gas recirculation
CO_2_: Carbon dioxide
CNG9: 7Natural gas case with a 97% substitution rate
LPPI: Low-pressure post-injection
LPI: Low-pressure injection
Rpm: Revolutions per minute
TDC: Top dead center
CFD: Computational fluid dynamics
℃A: Crank angle
HRR: Heat release rate
CH_4_: Methane
ATDC: After top dead center
Pmax: Maximum burst pressure
ITE: Indicated thermal efficiency
TE: Thermodynamic efficiency
IMEP: Indicated mean effective pressure
